# Differential responsiveness to BRAF inhibitors of melanoma cell lines BRAF V600E-mutated

**DOI:** 10.1186/s12967-020-02350-8

**Published:** 2020-05-11

**Authors:** Muna Al Hashmi, Konduru S. Sastry, Lee Silcock, Lotfi Chouchane, Valentina Mattei, Nicola James, Rebecca Mathew, Davide Bedognetti, Valeria De Giorgi, Daniela Murtas, Wei Liu, Aouatef Chouchane, Ramzi Temanni, Barbara Seliger, Ena Wang, Francesco M. Marincola, Sara Tomei

**Affiliations:** 1 Research Branch, Sidra Medical and Research Center, 26999 Doha, Qatar; 2 Department of Genetic Medicine, Weill Cornell Medical College in Qatar, Doha, Qatar; 3 Infectious Disease and Immunogenetics Section (IDIS), Department of Transfusion Medicine, Clinical Center, National Institutes of Health (NIH), Bethesda, USA; 4 Department of Biomedical Sciences, Section of Cytomorphology, University of Cagliari, Cagliari, Italy; 5 Institute of Medical Immunology, Martin Luther University Halle-Wittenberg, Halle/Saale, Germany; 6 Refuge Biotechnologies, Menlo Park, CA USA

**Keywords:** Next-generation sequencing, QPCR, Sanger sequencing, BRAF mutation

## Abstract

**Background:**

Most mutations in melanoma affect one critical amino acid on BRAF gene, resulting in the V600E substitution. Patient management is often based on the use of specific inhibitors targeting this mutation.

**Methods:**

DNA and RNA mutation status was assessed in 15 melanoma cell lines by Sanger sequencing and RNA-seq. We tested the cell lines responsiveness to BRAF inhibitors (vemurafenib and PLX4720, BRAF-specific and sorafenib, BRAF non-specific). Cell proliferation was assessed by MTT colorimetric assay. BRAF V600E RNA expression was assessed by qPCR. Expression level of phosphorylated-ERK protein was assessed by Western Blotting as marker of BRAF activation.

**Results:**

Three cell lines were discordant in the mutation detection (BRAF V600E at DNA level/Sanger sequencing and BRAF WT on RNA-seq). We initially postulated that those cell lines may express only the WT allele at the RNA level although mutated at the DNA level. A more careful analysis showed that they express low level of BRAF RNA and the expression may be in favor of the WT allele. We tested whether the discordant cell lines responded differently to BRAF-specific inhibitors. Their proliferation rate decreased after treatment with vemurafenib and PLX4720 but was not affected by sorafenib, suggesting a BRAF V600E biological behavior. Yet, responsiveness to the BRAF specific inhibitors was lower as compared to the control. Western Blot analysis revealed a decreased expression of p-ERK protein in the BRAF V600E control cell line and in the discordant cell lines upon treatment with BRAF-specific inhibitors. The discordant cell lines showed a lower responsiveness to BRAF inhibitors when compared to the BRAF V600E control cell line. The results obtained from the inhibition experiment and molecular analyses were also confirmed in three additional cell lines.

**Conclusion:**

Cell lines carrying V600E mutation at the DNA level may respond differently to BRAF targeted treatment potentially due to a lower V600E RNA expression.

## Background

Cutaneous melanoma is an aggressive disease representing one of the leading causes of mortality related to human cancers worldwide.

In the recent years, several molecular alterations have been identified as occurring during melanoma initiation and progression [1, 2]. The recognition of driving mutations in multiple melanoma oncogenes allowed the successful implementation of targeted therapies [3]. Perhaps, the most remarkable progress has been made after the identification of BRAF mutations in melanoma [4]. BRAF is a serine-threonine kinase, which transfers growth signals to the nucleus of the cells. More than 90% of BRAF mutations result in the valine to glutamic acid substitution (V600E) [4–6], associated with a 400-fold increased activity of the protein. Aside from BRAF mutations, NRAS mutations have also been described in about 15% of melanoma patients [7, 8], and result in the reduction of the intrinsic GTPase activity and in the constitutive activation of NRAS. Additionally, mutations in NF1 genes can lead to melanoma. The NF1 protein, neurofibromin 1, negatively regulates RAS proteins through GTPase activity. Melanomas with NF1 mutations typically occur on chronically sun-exposed skin or in older individuals, show a high mutation burden, and are wild-type for BRAF and NRAS [9, 10].

While the effects of the V600E mutation of BRAF have been extensively studied in experimental systems, several aspects of BRAF function remain poorly understood. Very few studies have investigated the role of BRAF expression in melanoma.

Major advances have been made in the clinical management of melanoma patients carrying BRAF mutations with the adoption of two BRAF specific inhibitors, namely vemurafenib and dabrafenib, approved by the Food and Drug Administration (FDA) in 2011 and 2013, respectively. The correct identification of cancer driving mutations is of paramount importance in cancer diagnostics as this allows the appropriate selection of target treatments and the implementation of personalized therapies [3]. To date, several methods are used in diagnostics to identify clinically relevant mutations. These methods include Sanger sequencing, immunohistochemistry, mutation-specific real-time polymerase chain reaction (q-PCR) and next generation sequencing (NGS) technologies [11–14]. Of note, BRAF mutation is usually detected at the DNA level, while BRAF expression is generally not assessed prior to target treatment selection.

When performing RNA-seq and Sanger sequencing on 15 melanoma cell lines, we found discordance between the results obtained by the two methods for three of the cell lines analyzed, specifically, these three cell lines showed a BRAF V600E mutation by Sanger sequencing but the mutation was not detected by RNA-seq. This prompted us to investigate whether this was due to a differential expression of BRAF WT and V600E alleles and whether these cell lines showed a different responsiveness to BRAF inhibitors as compared to the concordant WT and V600E mutant cell lines. We have tested three additional cell lines to confirm the data.

## Materials and methods

### Melanoma cell culture

Melanoma cell lines were derived from metastatic melanoma lesions from patients treated at the Surgery Branch, National Cancer Institute (NCI), Bethesda, MD and kindly donated by Dr Steven A. Rosenberg. All patients signed an informed consent approved by the Institutional Review Board of the National Cancer Institute. Two cell lines were purchased from ATCC, namely A375 and SKMEL28; one additional cell line, PIG1, immortalized from human melanocytes was kindly donated by Dr. Caroline Le Poole from Northwestern University, Chicago, Illinois, USA. These three cell lines were tested for validation purposes. Cells were cultured at 37 °C, in 5% CO_2_ with RPMI 1640 medium supplemented with 10% heat-inactivated FBS (Gemini Bioproducts), 0.01% GPS (penicillin, l-glutamine, streptomycin), and 0.01% fungizone (250 mg/L, Invitrogen).

### DNA isolation, BRAF and NRAS Sanger sequencing

Genomic DNA was isolated using QIAamp DNA Mini Kit (Qiagen, Germantown, MD) according to the standard protocol. DNA quality and quantity were estimated using Nanodrop (ThermoScientific, Pittsburgh, PA).

Each sample was screened for mutations in exons 11 and 15 of BRAF gene and exon 1 and 2 of NRAS gene. PCR was performed in a 20 µl final volume, containing 50 ng of genomic DNA, 10 µl of Qiagen HotStarTaq Master Mix Kit (Valencia, CA) and 500 nM of forward and reverse primers with the following cycling conditions: initial denaturation at 95 °C for 10 min; 35 cycles at 95 °C for 30 s, 56 °C for 30 s and 72 °C for 30 s; final step 72 °C for 10 min. Primers were selected using Primer3 software (http://frodo.wi.mit.edu/):

BRAF_ex11_F: 5′-TCCCTCTCAGGCATAAGGTAA-3′

BRAF_ex11_R: 5′-CGAACAGTGAATATTTCCTTTGAT-3′

BRAF_ex15_F: 5′-TCATAATGCTTGCTCTGATAGGA-3′

BRAF_ex15_R: 5′-GGCCAAAAATTTAATCAGTGGA-3′

NRAS_ex1_F: 5′-CACTAGGGTTTTCATTTCCATTG-3′

NRAS_ex1_R: 5′-TCCTTTAATACAGAATATGGGTAAAGA-3′

NRAS_ex2_F: 5′-ATAGCATTGCATTCCCTGTG-3′

NRAS_ex2_R: 5′-CACAAAGATCATCCTTTCAGAGA-3′

In each PCR reaction distilled water was used as a negative control. PCR products were purified with Exosap-IT (USB Corporation, Cleveland, OH) and labeled using Big Dye terminator kit v3.1 (Applied Biosystems, Foster City, CA). Excess dye terminators were removed using DyeEx 96 Kit columns following the manufacturer’s instructions (Qiagen). Sequencing was performed using Biosystems 3730 Genetic Analyzer (Applied Biosystems, Foster City, CA) and analyzed by Sequencher software (Genecodes, Ann Arbor, MI).

### RNA-Seq and data analysis

Total RNA was isolated from the cell lines using miRNeasy minikit (Qiagen) according to the manufacturer’s protocol. RNA quality and quantity were estimated using Nanodrop (Thermo Scientific) and Agilent 2100 Bioanalyzer (Agilent Technologies, Santa Clara, CA, USA). A A260/A280 ratio of ~ 2.0 was considered indicative of RNA of good purity. A RIN (RNA Integrity Value) of 7 was set as cut off for RNA quality. Only samples with RIN > 7 were used for further experiments. Enrichment in mRNA molecules was obtained by using oligo (dT) magnetic beads (Ambion^®^ Poly (A) Purist™ MAG Kit). After the mRNA was fragmented in short fragments (approximately 200 bp), cDNA was synthesized by random hexamer primers (Illumina TruSeq Stranded mRNA Library Prep Kit). The double-stranded cDNA was purified by QiaQuick PCR extraction kit (Qiagen) and went through an end repair process with the addition of a single ‘A’ base, and then ligation of the adapters. These products were then purified by agarose gel electrophoresis and enriched with PCR to create the final cDNA library. The library products were sequenced and 300 bp sequences were generated via the GAIIx Illumina sequencing platform. Raw reads were imported on a commercial data analysis platform CLC Genomics Workbench (CLC bio, MA, USA). An average of 20 million reads were generated for each sample run as per Illumina recommendations. Quality control checks on raw sequence data from each sample were performed using the QC analysis application tool. Adapter trimming was done to remove ligated adapter from 3′ end of the sequenced reads with only one mismatch allowed. After reads have been processed to meet a quality standard, they were aligned to the Human reference genome UCSC-Hg19, using the ultra high-throughput short aligner provided by CLC bio software. A Transcript Discovery analysis was performed to generate a transcript annotation file with an estimation of the relative abundances of each transcript by counting the number of reads that mapped to the genomic location of that transcript. Transcription level assessment has been obtained by the number of fragments per kilobase of transcript per million fragments mapped (RPKM).

### BRAF inhibitors treatment

The three discordant cell lines (MEL-2523, MEL-3025, MEL-3104) and the control WT (MEL-2805) and V600E (MEL-2492) cell lines were treated with BRAF inhibitors prior to running the proliferation assay and DNA and RNA mutational testing.

About 5 x 10^6^ cells were seeded in complete medium the day before treatment and incubated overnight at 37 ℃ with 5% CO_2_. Two BRAF-specific (PLX4720 and Vemurafenib) and one BRAF nonspecific inhibitor (Sorafenib) were purchased from Selleckchem, Houston TX and used for the inhibition treatment. Final concentration of PLX4720, Vemurafenib and Sorafenib were respectively 1 μM, 2 μM and 5 μM. Upon treatment, cells were used for DNA and RNA isolation (see below). Each experiment was done in triplicate. The same experiments were repeated on three additional cell lines for reproducibility, namely A375, SKMEL28 and PIG1. A375 and SKMEL28 are commercially available cell lines carrying the BRAF V600E mutation. PIG1 is a cell line transformed from normal epidermal melanocytes.

### MTT proliferation assay

The MTT assay is a colorimetric test used to assess the cell metabolic activity [15]. The NAD(P)H-dependent oxidoreductase enzymes constitutively present in the cells are able to reduce the tetrazolium dye MTT 3-(4,5-dimethylthiazol-2-yl)-2,5-diphenyltetrazolium bromide to formazan (an insoluble form), which is purple in color. The acquisition of color changes is the basis of this colorimetric assay. The cytotoxic effects of each inhibitor were tested using the MTT proliferation assay. About 5 × 10^3^ cells in 100 µl of complete medium were seeded into each well of a 96-well tissue culture plate and incubated overnight at 37 ℃ with 5% CO_2_. Cells were treated with indicated inhibitors in serum-free medium for a period of 24 h, 48 h and 72 h. Each treatment condition was done in triplicate. Then 20 µl of MTT (5 mg/ml) were added to each well and plates were further incubated for 4 h at 37 ℃. The formed crystals were solubilized by addition of 150 µl of DMSO in each well and cells agitated on an orbital shaker for 20 min. The optical density was measured using a spectrophotometer at a wavelength of 570 nm. The BRAF inhibition experiment was repeated at least three times, in which each cell line was assessed in triplicate.

### RNA and DNA isolation after inhibition treatment

Total RNA from the 15 cell lines was extracted using miRNeasy minikit (Qiagen) according to the manufacturer’s protocol. RNA quality and quantity were estimated using Nanodrop (Thermo Scientific) and Agilent 2100 Bioanalyzer (Agilent Technologies, Palo Alto, CA). A RIN of 7 was set as cut off for RNA quality. Only samples with RIN > 7 were used for further experiments. First- and second-strand cDNA were synthesized from 300 ng of total RNA according to manufacturer’s instructions (Ambion WT Expression Kit).

Genomic DNA was isolated using QIAamp DNA Mini Kit (Qiagen, Germantown, MD) according to the standard protocol. DNA quality and quantity were estimated using Nanodrop (ThermoScientific, Pittsburgh, PA). Only DNA of A260/A280 of ~ 1.8 were processed for downstream analyses.

### BRAF allele-specific PCR

Allele specific PCR was performed to detect BRAF V600E mutation and wild type BRAF sequence. Two PCR reactions (BRAF wild-type and BRAF V600E specific, respectively) were performed for each sample. For the RNA allele-specific PCR, the reverse primer was used for both the PCR reactions and designed spanning exon 15 and exon 16. The forward primers were designed to possess two bases substitution at 3′-end compared to wild-type sequences. These primers were, respectively:

BRAF15_R: 5′-GATGACTTCTGGTGCCATCC-3′

BRAF15_WT_F: 5′-TAGGTGATTTTGGTCTAGCTACAGT-3′

BRAF15_V600E_F: 5′-GGTGATTTTGGTCTAGCTACAAA-3′

For the DNA PCR, primers were designed as previously reported [16].

The PCR reaction was performed in a 25 μL final volume, containing 200 nM of forward and reverse primers, 2 μL of cDNA, 12.5 μL of 2× GoTaq MasterMix (Promega, Madison, WI, USA), which included BRYT Green as intercalating dye. In each reaction 0.3% HiDi formamide (Life Technologies, Grand Island, USA) was added to increase primer-annealing specificity. Cycling conditions were as following: initial denaturation at 95 °C for 10 min; 40 cycles at 95 °C for 15 s, 60 °C for 1 min. Reactions were run on a 7500 Fast Real-Time machine (Applied Biosystem, Carlsbad, CA, USA). To verify primers specificities, melting curves were generated at the end of PCR reaction. Fluorescent data were acquired during the extension phase. After 40 cycles, a melting curve for each gene was generated by increasing the temperature from 60 °C to 95 °C (1 °C for each step), while the fluorescence was measured. For each experiment a no-template reaction was included as a negative control. The qPCR data was analyzed using the 2^-(delta Ct)^ method and GAPDH as housekeeping gene.

### Extraction of protein and Western Blotting after inhibition treatment

Cells were harvested in a cell lysis buffer containing 20 mM Tris, pH 7.4, 1% Triton X-100, 40 mM NaF, 2 mM EDTA, 1 mM EGTA, 1 µg each of pepstatin, leupeptinand aprotinin, 1 mM PMSF, 1 mM NaVO4, 50 mM β-glycerophosphate, 40 mM p-nitrophenyl phosphate, and 1 mM DTT. The cell lysates were cleared of insoluble material by centrifugation at 14,000 g for 10 min at 4 ℃. The protein quantity was estimated by BCA Protein Assay Kit (Pierce Biotechnology, Waltham, MA, United States). Approximately 50 µg of protein was used in Western blotting. The Western blotting protocol was similar as described previously [17]. Equal loading was confirmed using β-actin antibodies. Western Blot experiment was repeated twice.

### Statistical analyses

Paired two-tailed Student’s *t*-test was used to determine differences of cell lines’ responsiveness to the inhibitors at day 3. ANOVA (analysis of variance) test was used to evaluate the effects of BRAF inhibitors. All the analyses were performed using Statgraphics Centurion (V. 15, StatPoint, Inc.).

## Results

Eight out of fifteen (53%) cell lines harbored the BRAF V600E mutation. Among the seven cell lines that did not display BRAF mutation, six cell lines harbored activating mutation in NRAS gene (four cell lines displayed Q61R, two cell lines displayed Q61K and Q61L respectively). BRAF and NRAS mutations were mutually exclusive in the cell lines tested.

RNA isolated from the 15 cell lines was tested by RNA-seq. Discordance between Sanger sequencing and RNA-seq was observed for BRAF but not for NRAS mutation assessment. Table 1 shows the concordance of BRAF mutation assessment between Sanger sequencing and RNA-seq.

**Table 1:** Assessment of BRAF mutation by Sanger sequencing and RNA-seq

	BRAF status	
Samples	Sanger sequencing	RNA-seq
MEL-1866	WT	WT
MEL-2035	V600E	V600E
MEL-2075	WT	WT
MEL-2155	WT	WT
MEL-2224	V600E	V600E
MEL-2427	WT	WT
MEL-2448	V600E	V600E
MEL-2458	V600E	V600E
MEL-2492	V600E	V600E
*MEL-2523*	*V600E*	*WT*
MEL-2744	WT	WT
MEL-2805	WT	WT
*MEL-3025*	*V600E*	*WT*
*MEL-3104*	*V600E*	*WT*
MEL-3107	WT	WT

Cell lines in italic are discordant for BRAF mutation assignment

*WT* wild type

Out of the fifteen melanoma cell lines tested, twelve cell lines showed concordant results between Sanger sequencing and RNA-seq. Three cell lines, namely MEL-2523, MEL-3025 and MEL-3104, were mutated by Sanger sequencing but wild type by RNA-seq. Of note, there were no cell lines in which the mutation was detected only by RNA-seq but not by Sanger sequencing. Thus, we hypothesized that in these three “discordant” cell lines the wild type allele was the only one to be expressed at the RNA level.

To test this hypothesis, we assessed the BRAF mutational status at the RNA level by allele specific qPCR. The three discordant cell lines resulted mutated by RNA allele specific qPCR, consistent with the Sanger sequencing data. This suggested that the mutated allele was also expressed at the RNA level in contrast to our hypothesis.

A closer look to the RNA-seq data (Table 2) showed that the RNA expression of the cell lines BRAF mutated had an overall low expression of BRAF gene, this low BRAF expression was marked in the three discordant cell lines and was in favor of the WT allele in two of the discordant cell lines. On the contrary, NRAS expression was consistently higher than BRAF expression in all cell lines tested.

**Table 2:** RNA-seq experiment

Cell line ID	BRAF WT	BRAF MT	BRAF total reads	NRAS total reads
MEL-2035	1	2	3	123
MEL-2224	14	16	30	150
MEL-2448	5	18	23	93
MEL-2458	4	6	10	69
MEL-2492	–	4	4	77
*MEL-2523*	*–*	*2*	*2*	*83*
*MEL-3025*	*5*	*2*	*7*	*78*
*MEL-3104*	*5*	*2*	*7*	*65*

Number of reads of BRAF and NRAS gene. Cell lines in italic are discordant for BRAF mutation assignment

**Fig. 1:**
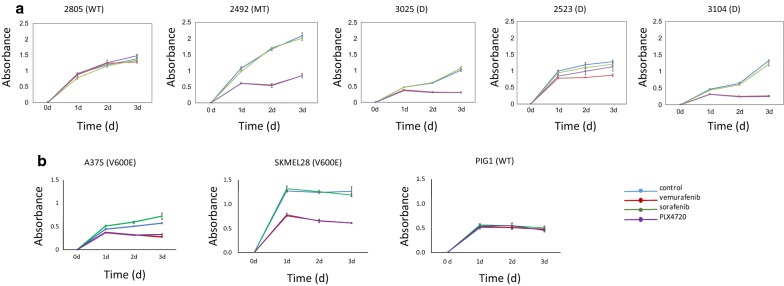
Proliferation curves of the five cell lines tested in this study and treated with BRAF inhibitors. WT, wild type. D, discordant. **a** Proliferation curves of the three additional cell lines assessed in the study for reproducibility. **b** Error bars indicate standard errors

We then postulated that a low expression of BRAF gene may translate in a lower responsiveness of melanoma cell lines to BRAF specific inhibitors. To test this hypothesis, we evaluated the cytotoxicity of BRAF inhibitors to the discordant cell lines by MTT proliferation assay. We have tested two specific BRAF inhibitors, namely vemurafenib and PLX4720 on the three discordant cell lines and on the two control cell lines (WT and V600E, respectively) whose mutation assignment was concordant between RNA-seq and Sanger sequencing (Fig. 1). We have used sorafenib, a non-specific BRAF inhibitor, as negative control. As expected, we found that the control WT cell line did not respond to any inhibitor while the control V600E cell line did respond to the BRAF specific inhibitors (vemurafenib and PLX4720) but did not respond to the non-specific inhibitor sorafenib (Fig. 1). The three discordant cell lines showed lower responsiveness to the two BRAF specific inhibitors vemurafenib and PLX4720 as compared to the control V600E cell line, especially cell line MEL-2523. The statistical differences between treatments at time point 3d (day 3) are reported in Table 3. P-values were obtained by two-tailed paired Student’s *t*-test. ANOVA test was also performed to evaluate the combined effect of the BRAF inhibitors (Additional file 1: Table S1).

**Table 3:** Statistical differences between treatments at time point 3d (day 3)

Cell line	Vemurafenib vs control	PLX4720 vs control	Sorafenib vs control
MEL-2805	P = 0.093	P = 0.384	P = 0.343
MEL-2492	*P = 0.044*	*P = 0.050*	P = 0.927
MEL-3025	*P = 0.003*	*P = 0.010*	P = 0.290
MEL-2523	P = 0.087	P = 0.220	P = 0.369
MEL-3104	P = 0.069	P = 0.067	P = 0.938

Statistically significant P-values are in italics (P ≤ 0.05)

P-values were obtained by two-tailed paired Student’s *t*-test

We have further explored the responsiveness to BRAF inhibitors in three additional cell lines, namely A375, SKMEL28 and PIG1. The mutation analysis revealed V600E mutation for both A375 and SKMEL28 while PIG1 was homozygous V600 wild-type. Consistent with the assumption that BRAF mutated cell lines respond to BRAF-specific inhibitors, A375 and SKMEL28 showed responsiveness to PLX4720 and vemurafenib but they did not respond to the non-specific inhibitor. Conversely, PIG1 proliferation curves did not show differences across all the inhibitor conditions, suggesting that the BRAF specific inhibitors had no effect on PIG1 (Fig. 1b).

**Fig. 2:**
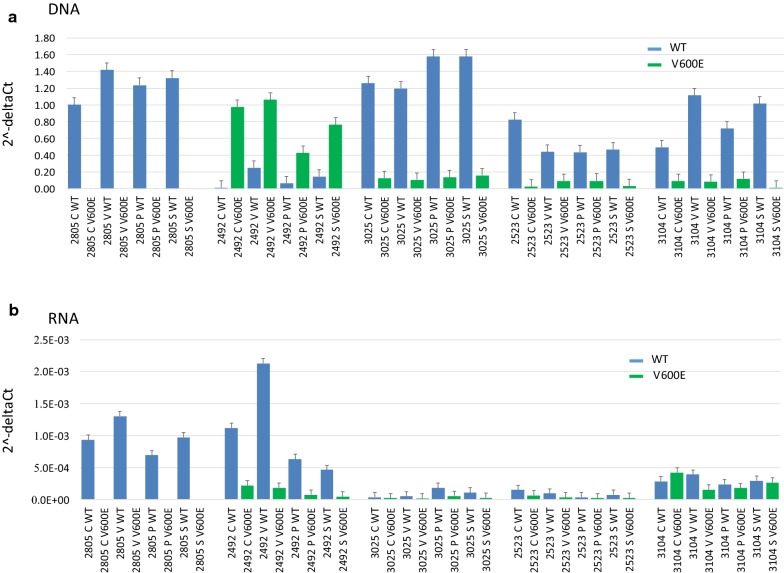
Quantitative allele-specific PCR on the DNA **a** and RNA **b** of the cell lines treated with BRAF inhibitors. (blue refers to WT allele, green refers to V600E allele). Error bars indicate standard errors. C: control. V: Vemurafenib. P: PLX4720. S: Sorafenib

**Fig. 3:**
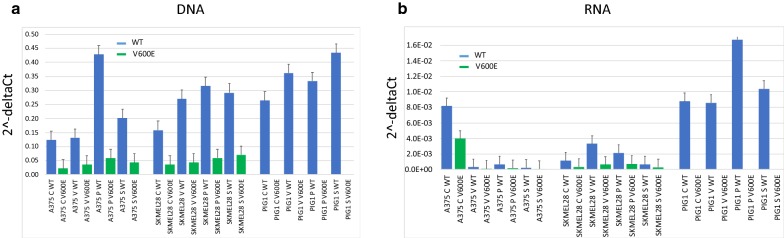
Quantitative allele-specific PCR on the DNA **a** and RNA **b** of A375, SKMEL28 and PIG1 cell lines treated with BRAF inhibitors. (blue refers to WT allele, green refers to V600E allele). Error bars indicate standard errors. C: control. V: Vemurafenib. P: PLX4720. S: Sorafenib

By using qPCR we also assessed the DNA abundance and RNA expression of both BRAF WT and BRAF V600E in all five cell lines after 2-h treatment with the BRAF inhibitors. As expected, the WT control cell line showed only BRAF WT amplification at both the DNA and RNA level. The control V600E cell line and the three discordant cell lines showed both BRAF WT and V600E amplifications at the DNA and RNA levels. Interestingly, the control V600E cell line showed an inverse trend of the WT versus the V600E form between DNA and RNA, specifically BRAF V600E was most represented at the DNA level as compared to BRAF WT, but BRAF V600E expression was lower as compared to the BRAF WT, suggesting the existence of regulatory mechanisms that may favor the expression of the WT allele even in the presence of a lower copy number at the DNA level. Once again, the three discordant cell lines overall showed a lower expression of BRAF (of both the WT and V600E alleles) as compared to the control WT and V600E cell line (Fig. 2a, b). The qPCR experiment was also performed on the three publicly available cell lines, PIG1, A375 and SKMEL28. As expected, the WT PIG1 cell line showed BRAF WT amplification at both the DNA and RNA levels, while the mutated cell lines A375 and SKMEL28 showed both BRAF WT and V600E amplifications at the DNA and RNA levels (Fig. 3). The WT form was consistently more abundant than the V600E form for both A375 and SKMEL28 cell lines at the DNA and RNA levels. Interestingly the BRAF RNA expression of PIG1 was overall higher as compared to the two BRAF V600E mutant cell lines, suggesting the existence of a possible regulatory mechanism that may decrease BRAF RNA expression in the presence of BRAF V600E mutation.

**Fig. 4:**
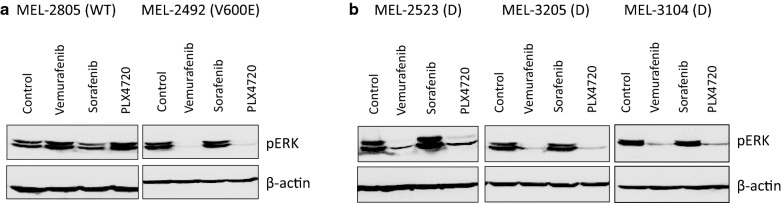
Western Blotting of p-ERK on the control cell lines **a** and the discordant cell lines **b**. WT, wild type. Equal loading was confirmed using β-actin antibody

We have tested the expression of phosphorylated-ERK (p-ERK) protein as marker of BRAF activation, and consequently of MAPK pathway activation. Western Blot analysis revealed a decreased expression of p-ERK protein in the BRAF V600E control cell line and in the discordant cell lines upon treatment with BRAF-specific inhibitors (Fig. 4). Overall, the discordant cell lines showed a lower responsiveness to BRAF inhibitors when compared to the BRAF V600E control cell line.

## Discussion

The assessment of the sensitivity and specificity of mutation detection of different methods is particularly relevant in the diagnostic setting. As an example, the identification of BRAF V600E mutation is important to indicate which melanoma patients will likely respond effectively to vemurafenib treatment [18, 19]. Other studies have revealed thousands of genomic regulatory regions possibly associated to responsiveness to BRAF inhibitors. Verfaillie and colleagues, for instance, have identified SOX10/MITF and AP-1/TEAD as regulators of proliferation and invasiveness and showed that knockdown of TEAD establishes a causative link between these transcription factors and sensitivity to MAPK inhibitors [20].

Since the discovery of BRAF mutations in melanoma by Davies and colleagues in 2002 [4], several studies have been performed to understand their association with different melanoma phenotypes and survival. However, all previous studies aiming at analyzing clinic-pathological associations with BRAF mutations have used DNA-based tests for the detection of the mutations. The significance of BRAF expression in relation to the responsiveness to BRAF inhibitors has still to be investigated thoroughly. A study by Birkeland and colleagues [21] has demonstrated that low BRAF and NRAS expression levels are associated with clinical benefit from dacarbazine treatment, however none of the subjects recruited in this study was treated with BRAF inhibitors.

Here we aimed at assessing whether a lower overall BRAF mRNA expression may correlate with a lower responsiveness to BRAF inhibitors. To our knowledge this question has not been previously investigated. We found here that three cell lines whose mutation assignment was discordant from the DNA and RNA levels and that express very low level of BRAF mRNA respond less to BRAF V600E specific inhibitors.

The response to BRAF inhibitors can be very different from patient to patient [19, 22]. A minority of BRAF V600E melanoma patients (3–5%) have no response. As the mutation assignment is performed at the DNA level, patients that do not respond to BRAF inhibitors may express low levels of BRAF mRNA while having the mutation at the DNA level. We believe that our findings may carry clinical relevance and should be validated in additional studies.

Two studies have assessed BRAF protein expression in melanoma [23, 24]. The first study by Wilmott and colleagues [24] has examined the predictive value of BRAF expression and response to BRAF inhibitors but did not find any association; a possible explanation of the lack of association may be the small cohort size or may relate to the low sensitivity of the immunohistochemistry technique that was used to assess BRAF protein expression. The second study by Hugdahl and colleagues [23] has shown that BRAF V600E expression in primary melanoma is associated with reduced survival, however BRAF expression was not assessed in relation to BRAF inhibitor treatment, additionally the samples recruited in this study were primary melanoma; the pathophysiological processes may differ between primary tumors and tumor metastases and the conclusions in studies focused on primary tumors may not necessarily apply to studies focused on tumor metastases.

Additionally, the issue of intratumoral heterogeneity has not been assessed in this study, we are planning to investigate whether the presence of different clones in the same cell line may account for the discordant mutation assignment.

## Conclusion

We show here that a low BRAF mRNA expression may explain a lower responsiveness to BRAF inhibitors in melanoma cell lines. These finding should be validated in additional studies with cohorts of a bigger size and employing tumor tissues rather than cell lines.

## Supplementary information

**Additional file 1: Table S1**. ANOVA test between treatments at time point 3d (day 3).

## Data Availability

The data presented here can be made available from the corresponding author on reasonable request.

## Abbreviations

FDA: Food and drug administration
MAPK: Mitogen-activated protein kinase
MTT: 3-(4,5-dimethylthiazol-2-yl)-2,5-diphenyl tetrazolium bromide
NGS: Next-generation sequencing
PCR: Polymerase chain reaction
WT: Wild type
