# Supplementary material for: Differential responsiveness to BRAF inhibitors of melanoma cell lines BRAF V600E-mutated
Source: J Transl Med. 2020 May 11;18:192. doi: 10.1186/s12967-020-02350-8 (PMC7216681; doi:10.1186/s12967-020-02350-8)
Supplement: Supplementary file 1 — Additional file 1: Table S1. ANOVA test between treatments at time point 3d (day 3). [file 12967_2020_2350_MOESM1_ESM.docx]

**Supplementary Table 1**. ANOVA test between treatments at time point 3d (day 3). The f-ratio value is 3.75372. The p-value is .032453. The result is significant at p < .05

|  | Control | Vemurafenib | PLX4720 | Sorafenib | Total |
| --- | --- | --- | --- | --- | --- |
| N | 5 | 5 | 5 | 5 | 20 |
| ∑X | 6.79 | 3.54 | 3.77 | 6.77 | 20.87 |
| Mean | 1.358 | 0.708 | 0.754 | 1.354 | 1.044 |
| ∑X2 | 9.7779 | 3.2464 | 3.8119 | 9.6827 | 26.5189 |
| Std.Dev. | 0.3732 | 0.4301 | 0.4923 | 0.3592 | 0.4995 |

| **Result Details** |  |  |  | |  |
| --- | --- | --- | --- | --- | --- |
| Source | SS | df | | MS |  |
| Between-treatments | 1.9585 | 3 | | 0.6528 | F = 3.75372 |
| Within-treatments | 2.7826 | 16 | | 0.1739 |  |
| Total | 4.7411 | 19 | |  |  |
